# A Wireless Sensor Enabled by Wireless Power

**DOI:** 10.3390/s121216116

**Published:** 2012-11-22

**Authors:** Da-Sheng Lee, Yu-Hong Liu, Chii-Ruey Lin

**Affiliations:** 1 Department of Energy and Refrigerating Air-Conditioning Engineering, National Taipei University of Technology, No. 1, Sec. 3,Chung-Hsiao E. Rd., Taipei 106, Taiwan; 2 Institute of Mechatronic Engineering, National Taipei University of Technology, Taipei 106, Taiwan; E-Mails: mayin6400@yahoo.com.tw (Y.H.L.); crlin@ntut.edu.tw (C.R.L.)

**Keywords:** wireless sensor, wireless power, chip-type sensor, UHF RFID reader, wireless sensor network

## Abstract

Through harvesting energy by wireless charging and delivering data by wireless communication, this study proposes the concept of a wireless sensor enabled by wireless power (WPWS) and reports the fabrication of a prototype for functional tests. One WPWS node consists of wireless power module and sensor module with different chip-type sensors. Its main feature is the dual antenna structure. Following RFID system architecture, a power harvesting antenna was designed to gather power from a standard reader working in the 915 MHz band. Referring to the Modbus protocol, the other wireless communication antenna was integrated on a node to send sensor data in parallel. The dual antenna structure integrates both the advantages of an RFID system and a wireless sensor. Using a standard UHF RFID reader, WPWS can be enabled in a distributed area with a diameter up to 4 m. Working status is similar to that of a passive tag, except that a tag can only be queried statically, while the WPWS can send dynamic data from the sensors. The function is the same as a wireless sensor node. Different WPWSs equipped with temperature and humidity, optical and airflow velocity sensors are tested in this study. All sensors can send back detection data within 8 s. The accuracy is within 8% deviation compared with laboratory equipment. A wireless sensor network enabled by wireless power should be a totally wireless sensor network using WPWS. However, distributed WPWSs only can form a star topology, the simplest topology for constructing a sensor network. Because of shielding effects, it is difficult to apply other complex topologies. Despite this limitation, WPWS still can be used to extend sensor network applications in hazardous environments. Further research is needed to improve WPWS to realize a totally wireless sensor network.

## 1. Introduction

Wireless sensor networks (WSNs) have shown great potential in industrial and commercial applications. For industrial applications that require real-time feedback control systems, such as packaging, manufacturing, wood machining, or plastic extrusion, a WSN can achieve cost reductions. Data exchange through a sensor network is suitable for smart sensors, thanks to their network interface. The advantages of a distributed architecture are numerous, and include increased flexibility, improved performance, predictive maintenance, simple installation, and cabling cost reduction [1]. For commercial applications, WSNs have been distributed in convenience stores in Taiwan for thermal comfort and energy conservation control. A case study conducted for three years showed that the convenience stores achieved marginal energy conservation and energy savings of up to 53%, recovering all investment in approximately 5 months [2]. This short cost-recovery period confirms that a WSN is a high value product for achieving energy conservation and a comfortable environment.

Although WSNs have great commercial potential, the problem of supplying power to the sensor nodes hinder their development. Using batteries for wireless sensors provides limited energy to perform demanding tasks, and how to maximize operation lifetime and achieve optimal resource management remains a challenge [3]. Low battery capacity causes node malfunctions and breaks the network, and this type of WSN needs regular maintenance and battery replacement. This reduces the reliability of the WSN and increases costs. Moreover, replacing batteries introduces pollution to the environment [4].

Charging sensor nodes remotely by an electromagnetic (EM) wave is a novel idea for WSNs. Wireless charging was first demonstrated by Nicola Tesla at the end of the nineteenth century [5], illuminating wireless lamps using energy sources coupled to them through an alternating electric field. Tesla announced that a Tesla tower, a large coil lighting fixture for a hall or room, could be moved and put anywhere without being electrically connected to anything. Although Tesla was a man before his time, this type of imaging power supply structure has not been implemented in practice.

In 2007, a research team at the Massachusetts Institute of Technology (MIT) demonstrated wireless power transfer over a 2 m distance, from a coil on the left to a coil on the right, powering a 60 W light bulb [6]. They used a key technology development, called magnetically coupled resonance, to increase wireless charging efficiency. This design is an important step toward accomplishing wireless power in the future. However, their prototype requires two huge coil antennas of approximately 60 cm in diameter for transmitting and receiving power. This is too big to be used for real applications, such as a small sensor node.

From 2008 till now, wireless charging technology began to develop rapidly, and many companies have launched commercial products on the market. The Wireless Power Consortium (WPC) held its first meeting on 18 December 2008, in Hong Kong. The WPC published the Qi low power specification in August 2009, 18 months after its first meeting, and certified the first Qi product in September 2009. NTT-Docomo announced the first mobile phones with integrated Qi receivers in May 2011 [7]. Commercial manufacturing techniques can effectively reduce the antenna size, making it possible to integrate wireless charging in a mobile phone or small handheld device. However, commercial techniques use inductive coupling at a high frequency (HF) of up to 100 kHz. An EM wave in the HF band has a long wavelength, and has difficulty propagating through space. Thus, it has a short charging distance of several millimeters to a few centimeters. For a wireless sensor network, the charging distance should exceed a meter or even several tens of meters. Thus, current commercial wireless charging products cannot be used to enable a wireless sensor node because of their limited driving distance.

Intel announced the Wireless Sensing and Identification Platform (WISP) in 2010 [8]. The WISP consists of small, battery-free devices that use radio frequency identification (RFID) technology to power themselves. Intel WISP features a wireless power supply, bidirectional communication, and an RFID sensor network consisting of multiple sensor devices and one or more readers. Commercial RFID readers employ an EM wave at an ultra-high frequency (UHF) band, ranging from 860 to 960 MHz, to enable tags to be distributed meters away. The UHF band wave has a short wavelength, reduced antenna size, and the ability to deliver power far away [9]. Using tags instead of sensors, a RFID sensor network has been demonstrated [10].

From its introduction as a Tesla tower to current advances in the WPC Qi standard and UHF RFID technology, wireless power supply is no longer only a theoretical concept: it has been realized. The WISP platform is currently the most promising architecture for constructing a wireless sensor powered wirelessly. However, the WISP depends on RFID technology, yet its RFID usage model is unlike that of a WSN. Two problems make it difficult to apply the WISP to real WSN applications. Based on the ISO 18000-6C standard [11], RFID communication includes power charging and data delivery stages. The variant power densities of the EM wave applied in two stages creates the first problem because the WISP loses power in the middle of a task. The second problem is that RFID communication is highly asymmetric in reading and writing tasks. This complicates protocols designed to gather and process sensor data. Indeed, the RFID sensor network demonstrated in [10] has an open and programmable RFID tag. This function cannot be compared with a real wireless sensor node.

This study presents a modified WISP platform and proposes a new architecture to overcome these problems. Based on the development of wireless charging technology, this study develops a wireless powered wireless sensor (WPWS) and tests if it can satisfy the following performance indices:

- Stability of harvesting energy from EM wave power: The WPWS should harvest energy from an EM wave stably and send back sensor signals to a wireless backend in a reasonable response time. The EM wave source uses a standard RFID reader that follows the air protocol regulated by ISO 18000-6C. There is no need to modify existing RFID equipment, which is well developed for wireless charging.
- Accuracy of WPWS sensors: The WPWS should send back sensing data with an acceptable packet loss rate. This ensures that the remote backend can decode signals and obtain sensing data as accurate as those obtained from laboratory instruments. However, obtaining the correct sensing data is the key to a wireless sensor node.
- Topology for constructing a sensor network: To construct a sensor network, the EM waves emitted from the RFID reader should enable sensor nodes in a distributed area. Multiple sensors can send back data simultaneously. This design is critical for constructing the physical layer of a sensor network. This feature may be similar to the interrogation zone in RFID equipment. This should enable a completely wireless sensor network, in which all wireless sensor nodes are enabled by wireless charging. However, it is still a challenge for a standard RFID reader because enabling a sensor node requires more power than querying a tag. The type of topology to use in a wireless charging network is still under investigation.

The following sections discuss the technical details of WPWS and an experimental setup to verify its performances.

## 2. WPWS Design Concept

**Figure 1.**
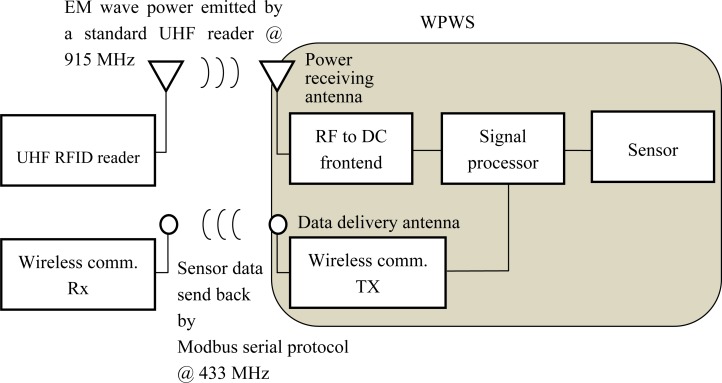
Dual antenna structure of a wireless powered wireless sensor (WPWS).

This section describes the design concept of a wireless sensor enabled by wireless power. A sensor node may take advantage of an RFID system that uses a UHF EM wave to deliver wireless power. However, data communication through the RFID protocol is unlike that of a sensor protocol. An RFID system works by querying static data such like a fixed identification number from a tag. A wireless sensor node must send back dynamic data such like variable data obtained from sensors. The different communication features of these two protocols make it difficult to accommodate both designs in the same system. This study proposes a dual antenna structure for a wireless sensor node to integrate the RFID and sensor protocols. This structure should have the advantages of both protocols and enables a WPWS. Figure 1 shows a schematic view of this type of system.

As Figure 1 shows, the WPWS harvests power from a UHF RFID reader. Following ISO 18000-6C regulations, the reader emits an EM wave at 915 MHz to enable remote sensor nodes. Using the power receiving one on the node, the EM wave power is drained to RF to the DC frontend. This frontend is a specially made power converter designed by our lab. It consists of a multi-stage rectifier, transmitter, and regulator. The multi-stage rectifier provides the initial RF to DC conversion. The transmit block can short or open the antenna using a switch to reflect or receive EM waves, respectively. Using switching control, the regulator can provide a constant DC power to the processor, sensor, and related circuits. The design concept of a wireless power supply consists of a power receiving antenna and an RF to DC frontend. That allows a wireless node to harvest energy from a remote RFID reader. The WPWS should meet the first performance requirement: stably harvesting energy from EM wave power.

The signal processor deals with input from the sensor and then sends it to the wireless communication module. The proposed design uses a commercial RF module working in the 433 MHz band, and adopts the Modbus serial protocol to construct a sensor protocol [12]. This provides a flexible structure that can be programmed for various sensor types. The data delivery antenna sends signals from the node, the wireless comm., Tx, to data receiving backend, the wireless comm., Rx. Using an independent antenna helps avoid interference between RFID communication and sensor signal communication. The WPWS should send back sensor data accurately to meet the second performance requirement: reporting data from the sensor accurately.

Though a Dual-Band system for remotely powered and wireless sensing has been proposed by Kopyt *et al.*[13], the completed prototype and experimental works only focused on single sensor driving but won’t be applied for constructing a sensor network. Till 2012, the nearest research and demonstrations still emphasize achieving a RF-powered, passive wireless sensor tag as a high-function RFID. The wireless powered wireless sensor can’t be used to form a wireless powered sensor network [14,15].

A sensor network requires the definition of three layers: the physical (PHY) layer, media access control (MAC) layer, and application (APP) layer [16]. The PHY layer provides an air protocol for communication. The proposed design integrates two working bands, 433 MHz and 915 MHz, to form a special PHY layer for the WPWS. The MAC layer primarily rules the coding and encoding algorithm. Through media adaption, sensor data can be sent back accurately for applications. This study adopts the Modbus serial protocol to construct the MAC layer for the WPWS. The APP layer provides definitions of application objects, ensures data paths, and helps construct the whole network. The Modbus protocol supports the APP object definitions. However, the wireless charging distance and charging area remain unclear, creating uncertain data paths. The data paths between wirelessly powered sensor nodes and battery-powered nodes makes nodes must be distributed in different topologies. Thus, it is necessary to determine how to construct a whole network using the WPWS and a suitable topology. After investigating the suitable topology and defining proper property of APP layer, the WPWS can meet the last performance requirement: construct a sensor network. This is the key topic of this study. If the WPWS can form a suitable topology, wireless power can enable a wireless sensor network to be complete wireless.

Based on the concepts discussed in this section, this study reports the design and fabrication of a WPWS prototype. The experiments in this study were conducted to the performance of the WPWS and to verify if it can meet the three performance indices discussed previously. Before introducing our experimental results, the following section provides detailed fabrication specifications and describes the completed WPWS prototype.

## 3. WPWS Prototype

Wireless power and sending return signals through wireless communication enables a completely wireless sensor network. This study reports the design and fabrication of a prototype based on the dual antenna structure discussed in Section 2. The following sections introduce the details of each functional block diagram of a WPWS (Figure 1).

### 3.1. Power Receiving Antenna

The proposed design uses a dipole antenna as the power receiving antenna. This is a wide-spread type of antenna for UHF RFID applications [9]. A dipole antenna can pick up a wide variety of radio frequency (RF) signals. This feature helps the WPWS effectively gather power from an EM wave. A dipole antenna has a simple construction. By combining two metal slots with a total length equal to half the wavelength of the carrier wave, the antenna can gather power from a remote EM wave. Although this antenna has a simple design, this study uses Ansoft HFSS simulation software to optimize it for power reception. This study also uses a 915 MHz frequency EM wave as the wireless power source. The basic length can be determined according to this frequency. However, with respect to different antenna widths, the length should be adjusted to achieve the lowest return loss, S_11_.

This study simulates a planar antenna printed on the FR4 circuit board. The material properties include board thickness, *h* = 1.6 mm; dielectric coefficient, *ɛ_r_* = 4.4; loss factor = 0.02; metal film thickness = 0.03 mm; conductivity = 5.8 × 10^7^ S/m. Based on these parameters, the effective dielectric coefficient can be calculated as follows:

(1) $${ɛ}_{\mathrm{eff}}=\frac{{ɛ}_{r}+1}{2}+\frac{{ɛ}_{r}-1}{2}.\left[\frac{1}{\sqrt{1+\frac{12\cdot h}{w}}}+0.04\cdot {\left(1-\frac{w}{h}\right)}^{2}\right]$$

where *w* is the width of a dipole antenna. This is under determined and can be adjusted to get the optimized design. The length of the dipole antenna should be adjusted according to *ɛ_eff_* using the following equation:

(2) $$L_{\mathrm{real}}=\frac{L_{\mathrm{Free}\;\mathrm{space}}}{\sqrt{{ɛ}_{\mathrm{eff}}}}=\frac{\lambda /2}{\sqrt{{ɛ}_{\mathrm{eff}}}}$$

where *L_real_* indicates the real length of the dipole antenna printed on FR4 board and *L_Free space_* represents the half-length of 915 MHz, as Equation (2) shows. For different settings of *w* and *L_real_*, this study calculates the S_11_ parameter using Ansoft HFSS to obtain the lowest value. The lowest return loss, S_11_, indicates the highest efficiency of harvesting power from the EM wave.

**Figure 2.**
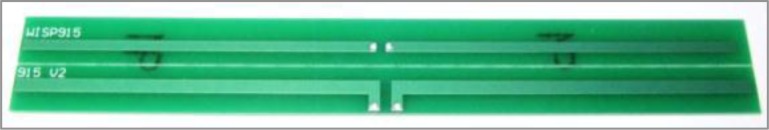
A 67.9 mm (L) × 5 mm (W) dipole antenna printed on FR4 board to serve as a power receiving antenna.

Results show that *L_real_* = 67.9 mm and *w* = 5 mm has the lowest S_11_ = −16.55 dB. Under this condition, the power receiving bandwidth is 10.03% of the central frequency. The resonance Q value is 9.97. This design can achieve a high power-receiving efficiency and wide bandwidth to harvest power in a broad frequency range, which is important for sensor applications under varying field conditions. The prototype was fabricated based on these parameters. Figure 2 shows a photograph of the power receiving antenna.

### 3.2. RF to DC Frontend

**Figure 3.**
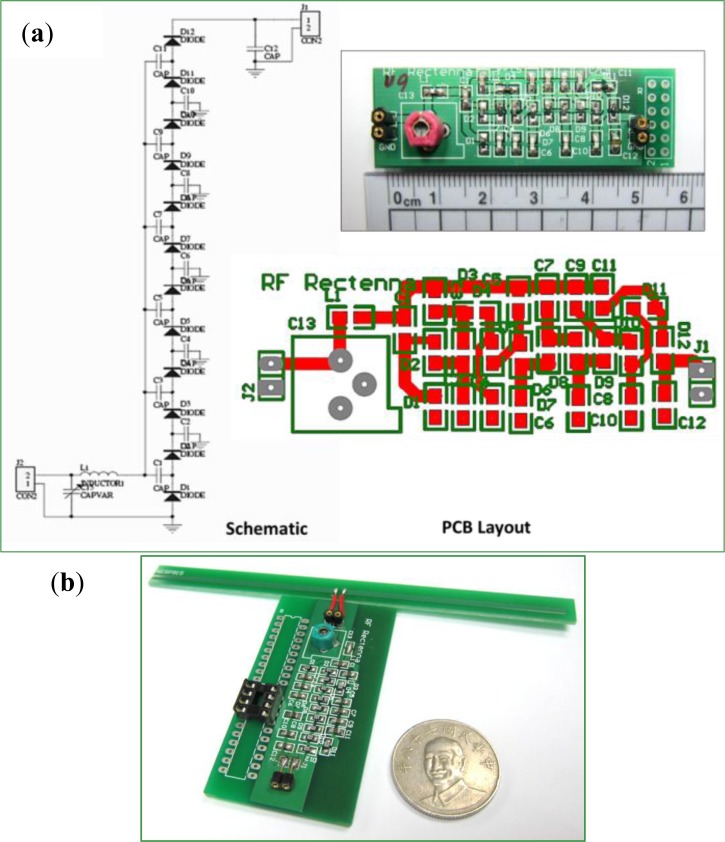
The RF to DC frontend converts EM wave power to DC current using a multi-stage rectifier: (**a**) Schematic view of circuit diagram, PCB layout and trial-fabricated sample. (**b**) Completed prototype.

A home-made RF to DC frontend as shown in Figure 3 converts EM wave energy to DC power to drive the following circuits. As discussed in Section 2, it consists of multi-stage rectifier, transmitter, and regulator. The rectifier consists of a resonant loop to match and input RF input, a decoupling capacitor, and a voltage rectifier. A Schottky diode rectifies the RF band energy. This study uses commercial components from Avago Tech, includingHSMS-282C/Y, HSMS-285C/Y, HSMS-286C/Y, to construct the rectifier. The transmitter either reflects or receives the EM wave using the short or open bypass switch of the power receiving antenna. The diode is non-linear device. If circuits have high loadings, the output will exhibit a ripple. A low pass filter eliminates this ripple, maintaining a constant DC voltage.

### 3.3. Signal Processor

The signal processor is the core of a sensor node. It responses to process data from sensor, codes the data, and sends it back to the remote data backend wirelessly. This study uses a PIC16F690 from Microchip co. as the signal processor on the node. This chip uses reduced instruction set computing (RISC) and supports dual bus data streaming, and has the feature of ultra low-power wake-up.

**Figure 4.**
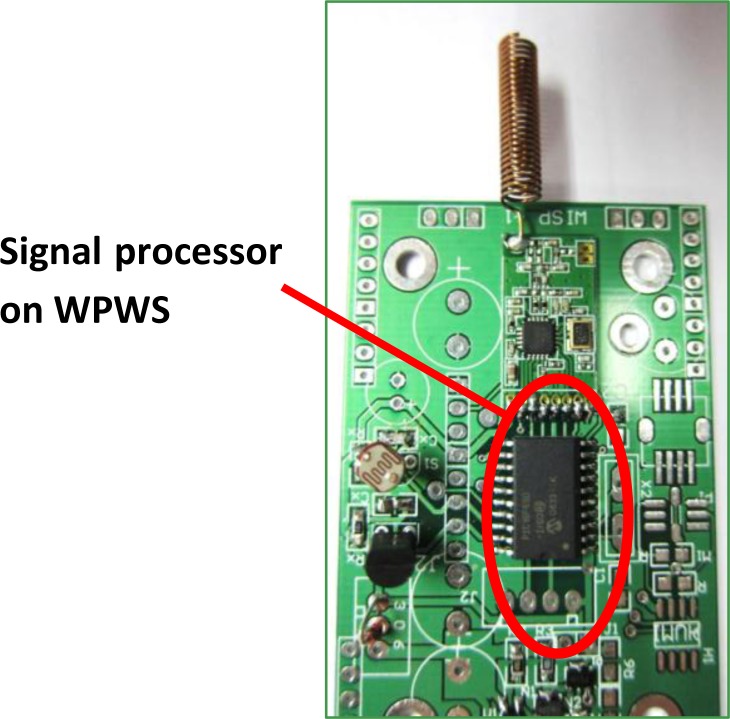
A commercial signal processor, the PIC17F690 chip, serves as the core of the sensor node.

This allows the signal processor to process working threads even under poor power receiving conditions. This is the key for realizing a wireless sensor node enabled by wireless power. Figure 4 shows a photograph of the sensor node controlled by the signal processor. Table 1 lists the detailed parameters of the PIC chip.

### 3.4. Wireless Communication Module and Data Delivery Antenna

The prototype in this study uses a commercial wireless communication module, CC1101 from TI Co. Ltd. This module is a paired module with a transmitter, Tx, on the sensor node, and receiver, Rx, on the remote data backend. Communication uses a carrier wave at 433 MHz. A helical antenna based on the ISO 18000-7 protocol serves as a data deliver antenna for communication. This study adopts the frequency shift key (FSK) method, regulated by the ISO standard, to encode data. Sensor data are processed by the signal processor and sent to the wireless module through the SPI. Sensor data are packed according to the Modbus protocol [17], which is a standard sensor protocol with a master/slave structure. One master node can support 247 slave node communications.

Each data communication packet consists of four blocks, including the address, function code, data, and error check blocks. The address block indicates where the message is from and where it will be delivered. The function code and data blocks store information about the sensor type and sensor data. The proposed protocol used two coding methods: ASCII and RTU. The RTU mode codes sensor data into communication packets. The maximum communication speed reaches 500 kbps. Using the error check and the feature function of CC1101—link quality indication (LQI), the packet loss rate can be lowered to ±1%.

**Figure 5.**
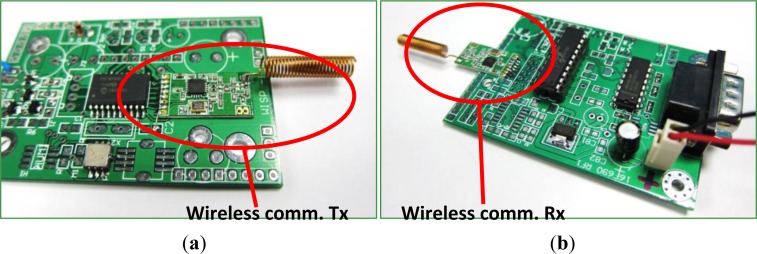
The commercial wireless communication module, the CC1101 module, used as a sensor node to send back sensor data. Paired modules are used to transmit data from the sensor node, Wireless comm. Tx as shown in (**a**), to data receiving backend, wireless comm. Rx as shown in (**b**).

With a high efficiency power amplifier, the communication distance can be as far as to 350 m. However, the communication speed and distance are still under investigation because wireless power driving may be ineffective at driving the sensor node. Figure 5 shows a photograph of the Tx on the sensor node, the Rx on the remote data receiving backend, and the attached helical antenna. Table 2 shows the main parameters of the CC1101 module.

### 3.5. Temperature and Humidity Sensor

**Figure 6.**
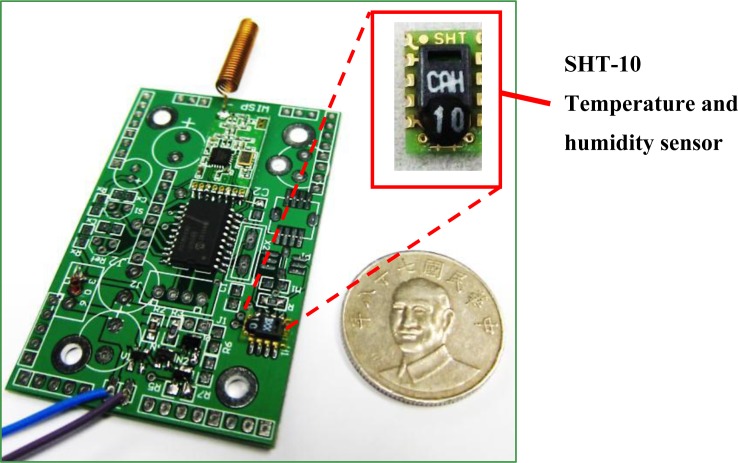
The temperature and humidity sensor module uses a chip-type sensor, SHT-10.

This study uses a chip-type sensor, SHT 10 from Sensiron, to detect temperature and relative humidity. This chip has a 14-bit ADC and can achieve accurate detection. For temperature sensing, the accuracy reaches ±0.5 °C in a 25 °C standard environment. Regarding relative humidity measurement, the accuracy reaches ±4.5% under conditions of 20%–80% relative humidity. The response time for gathering both types of data is less than 4 s. All detection data are directly encoded on the chip and sent to the signal processor through the SPI. Then delivering to wireless communication, data can be sent back to remote backend. The sensor module consists of a chip-type sensor, signal processor, and wireless communication module. Figure 6 shows a photograph of the temperature and humidity sensor module. Table 3 shows the parameters of the module.

### 3.6. Optical Sensor

**Figure 7.**
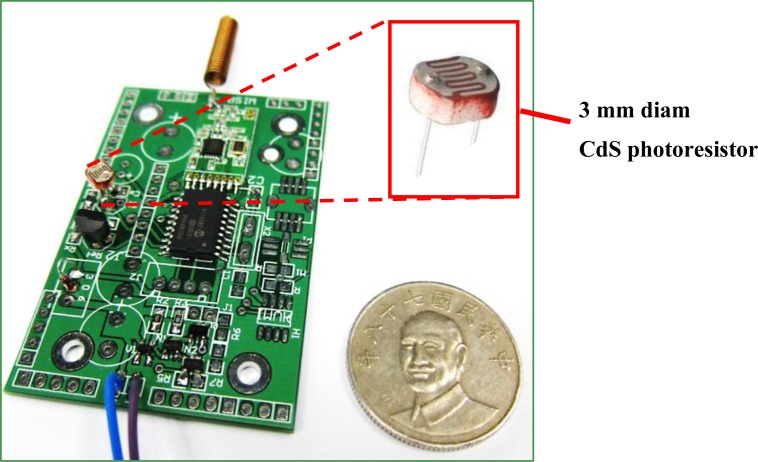
Using a CdS light sensor, the optical sensor module is designed and fabricated as shown in the following photograph.

A CdS photoresistor from Senba Co. with 3 mm diameter served as the optical sensor. This design uses a 10-bit ADC embedded in the signal processor to address the analog sensor signal and deliver it to the wireless module. The accuracy reaches ±0.5 lux in an artificial lighting environment. Figure 7 shows the complete optical sensor module, and Table 4 gives the module parameters.

### 3.7. Flow Sensor

**Figure 8.**
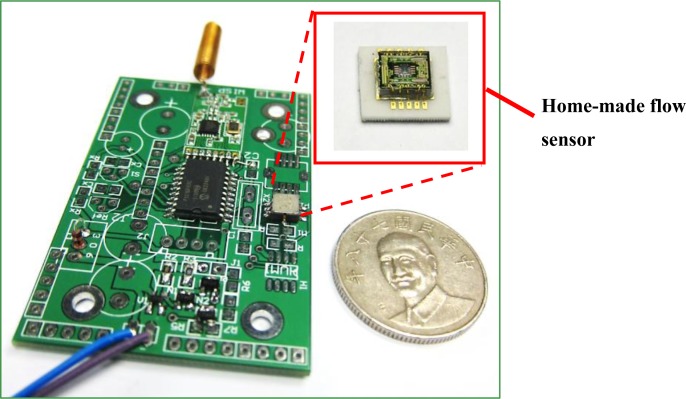
The flow sensor module uses a home-made flow sensor.

The proposed design uses a home-made flow speed sensor to detect air flow. This chip, with its microstructures and mixed signal processor, was fabricated based on standard 0.5 μm 2P2M CMOS process and the post-CMOS micromachining process. Previous research provides a detailed introduction to this sensor [18]. We adjusted the sensitivity of the chip to fit environmental applications, with a wind velocity ranging from 0.1 to 0.45 m/s. The accuracy can reach ±0.02 m/s. Because of this high sensitivity, the power consumption increased to 70 mW. Figure 8 shows the complete flow sensor module, and Table 5 gives the module parameters.

### 3.8. Completed Prototype

**Figure 9.**
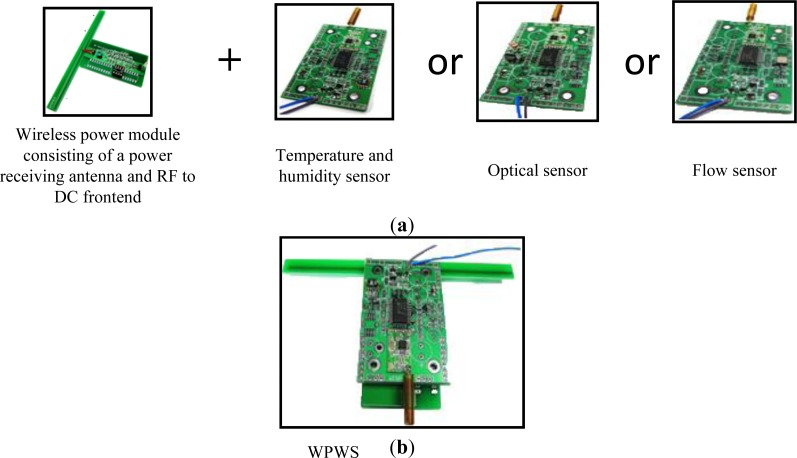
Combining wireless power module and each sensor module as shown in (**a**), the WPWS was fabricated for testing (**b**).

The wireless power module, consisting of a power receiving antenna and a RF to DC frontend, was fabricated to supply power for sensors. The final WPWS prototype combined a wireless power module and sensor module. The three sensor modules include temperature and humidity, optical, and flow sensors. Each sensor was connected to the WPWS individually to test its performance. Figure 9(a) shows the combination of three different sensor modules, whereas (b) shows a photograph of a whole WPWS prototype.

Figure 9(b) shows the dual antenna feature. The WPWS uses a 915 MHz band to harvest power and a 433 MHz band for communication, forming a special PHY layer. This structure offers the advantages of an RFID system and wireless sensor. The Modbus protocol facilitates master/ slave communication between the WPWS and the MAC layer. Object definition is also based on the Modbus protocol. However, whether the WPWS can form a topology with EM wave power from a standard RFID reader remains unclear, creating some uncertainties in the APP layer. Although the specifications described in this section can ensure that the WPWS works well as a wireless sensor, it is still not clear if the WPWS can be used to construct a sensor network. Thus, experiments are necessary to verify the working performance of the WPWS. The following section introduces experimental setups used for testing WPWS performance. These experiments not only checked if a wireless sensor can be effectively enabled by wireless power, but determined if the wireless sensor network can function with wireless power.

## 4. Experimental Setup

The WPWS prototype is enabled remotely by a standard RFID reader. A commercial reader, Alien ALR9900, was chosen as a wireless power source. This equipment can emit an EM wave in a UHF band ranging from 902.75 MHz to 927.25 MHz. The emitted power intensity can reach 4 W. This study selects a 915 MHz frequency and 1 W emitted power for testing. These two conditions meet local regulations in Taiwan. The wireless power performance was also tested under these regulated conditions.

A circular polarized antenna, ALR 9611-CR, was chosen to emit EM waves from the reader. This is the standard antenna matched to the Alien ALR9900, and provides an antenna gain of up to 5.73 dBi. A preliminary test shows its impedance is approximately 50 Ω. The dipole antenna on the WPWS has an impedance of approximately 70 + j40.5 Ω, and the antenna gain is 1.75 dB. The total antenna gain, including the reader and the WPWS, is 7.48 dB.

The experiments in this study did not test a vertical polarized antenna. For this type, the dipole antenna on the WPWS must be aligned to the reader antenna to harvest EM wave energy in a specified direction. The vertical antenna can deliver higher power than a circular polarized antenna. However, it is difficult to align sensor nodes to the reader direction. This limits the topology of the sensor network. Therefore, a vertical polarized antenna is not a good choice for the WPWS.

**Figure 10.**
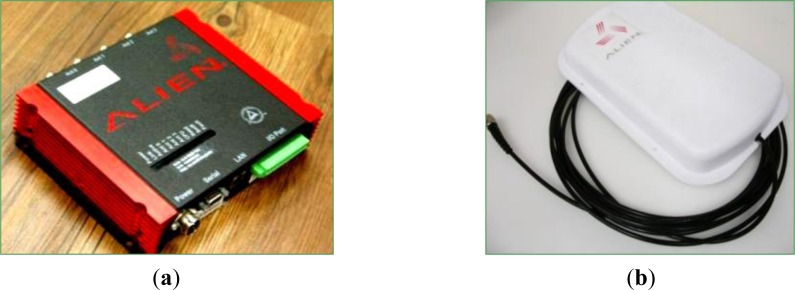
A standard UHF RFID reader (**a**) for emitting EM waves through a planar reader antenna (**b**) to enable a WPWS.

Figure 10(a) shows the RFID reader, which has dimensions of 20.32 cm (L) × 21.08 cm (W) × 4.58 (H) cm. One reader can support four antennas. Thus, multiple antennas can be used to construct a sensor network. Figure 10(b) shows the circular polarized antenna, which has dimensions of 28.4 cm (L) × 19.5 cm (W) × 4.3 cm (H). A spectrometer was used to monitor the EM wave power received by the WPWS. During performance tests, the WPWS was connected to the spectrometer. The peak dB value at 915 MHz was continuously recorded using a FFT tool.

**Figure 11.**
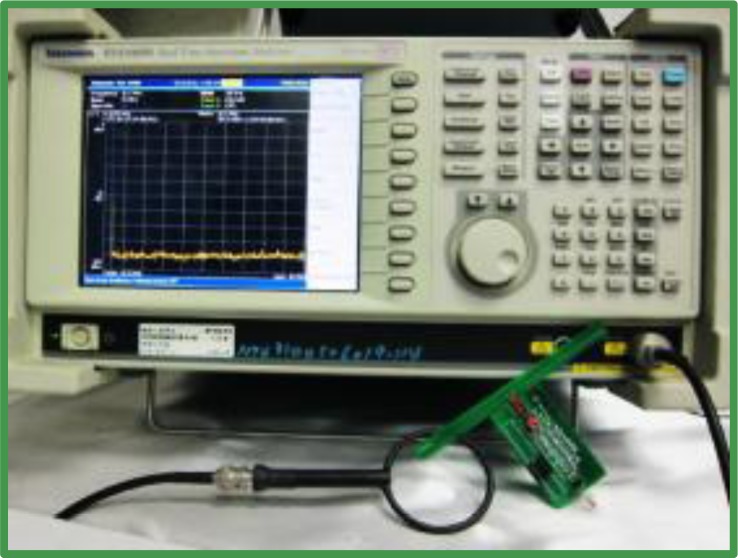
Tektronix RSA3408B spectrometer used for monitoring the intensity of the EM wave power received by the WPWS.

A dB value higher than the background noise indicates effective energy harvesting. The experiments in this study used a Tektronix RSA3408B spectrometer (Figure 11), which is capable of analyzing DC to 8 GHz frequency, to determine the stability of harvesting energy on the WPWS in the frequency domain. The key index of dB value at 915 MHz is reported.

**Figure 12.**
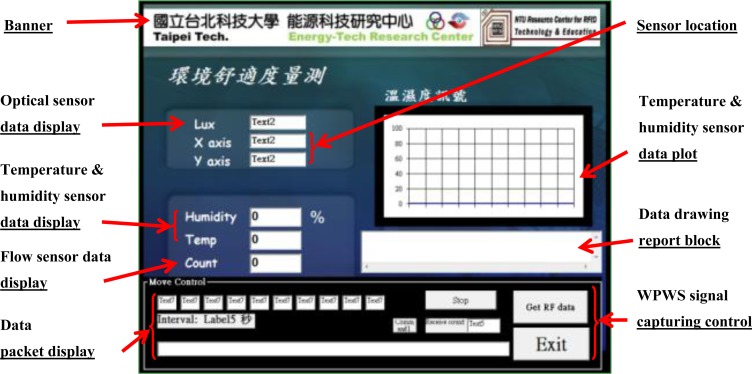
The software used to display sensing data, record communication packets, and evaluate the stability of the WPWS.

In addition to the frequency domain, this study also investigates working stability in the time domain. Using monitoring software developed by our lab, all data packets from the WPWS were recorded to evaluate communication status. As introduced in the WPWS prototype section, data packets were defined by the Modbus protocol. One data includes 4 bits sensor node code, 12 bits sensor data, 2 bits checksum, and 3 bits end of stream. After the wireless communication module, Rx, received the end of stream from the Tx, the checksum code was checked to verify if correct data was received. Using the LQI function of the wireless communication module, the packet loss rate can be suppressed to less than 1%. However, this produces a longer response time because the hardware limits the packet delivery if it has insufficient linking intensity. Unstable power harvesting on the WPWS causes the Tx side to work in a low power condition, increase the response time. This is another index for stability. Actually, the response time of the WPWS influences sensor performance. For environmental detection, the response time should be kept in second order to collect data of rapid variation. Figure 12 shows the data packet monitoring software, which can display sensing data and calculate response time automatically according to received data packet.

This study evaluates the working stability of the WPWS using hardware, the spectrometer shown in Figure 11, and the communication packet monitoring system shown in Figure 12. It investigates the response time of the WPWS equipped with different sensors to determine its working status. The received EM wave strength is used to evaluate received power intensity. With these two data sets, we discuss whether the WPWS can harvest energy stably from EM waves.

**Figure 13.**
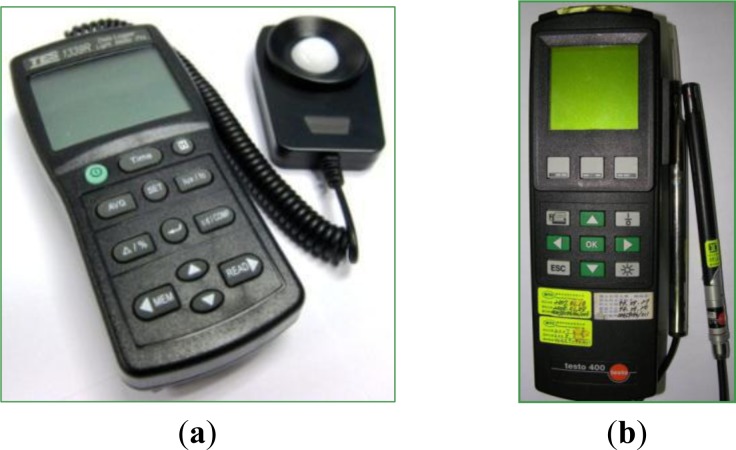
Laboratory equipment including an optical detector, TES 1339R, as shown in (**a**), and a multiple sensor module, TESTO 400, as shown in (**b**). These devices were used to verify the accuracy of a WPWS equipped with different sensor modules.

Accuracy is critical to the function of a sensor node. The sensor data sent to the software as shown in Figure 12 was compared with those obtained from laboratory equipment to illustrate sensor accuracy. This study uses temperature and humidity, optical, and air flow sensor modules for tests. Corresponding to the three types of WPWS, a Testo 400 multiple sensor m and TES1339R optical detector were used as comparative equipment to verify accuracy. The TES1339R detector is a professional optical detector corresponding to the local CNS 5119 class II standard. It has a detection range of 0.01–999,900 lux, with a resolution of 0.01 lux and accuracy of ±3%. The Testo 400 sensor with temperature and humidity probes can measure temperature ranges from −20 to 70 °C with solution of 0.1 °C and accuracy of ±0.4 °C at a range of −10 to 50 °C. To measure humidity, the range is within 0%–100% RH, resolution is 0.1%, and accuracy is ±2% RH at 2%–98% RH. The Testo 400 with hot ball anemometer can detect wind velocity from 0 to 10 m/s. The resolution is 0.01 m/s and the accuracy is ±0.03 m/s. All of these specifications are better than the chip-type sensors used in this study, and can therefore serve as a reference for comparative experiments. Figure 13(a,b) shows these devices, respectively.

**Figure 14.**
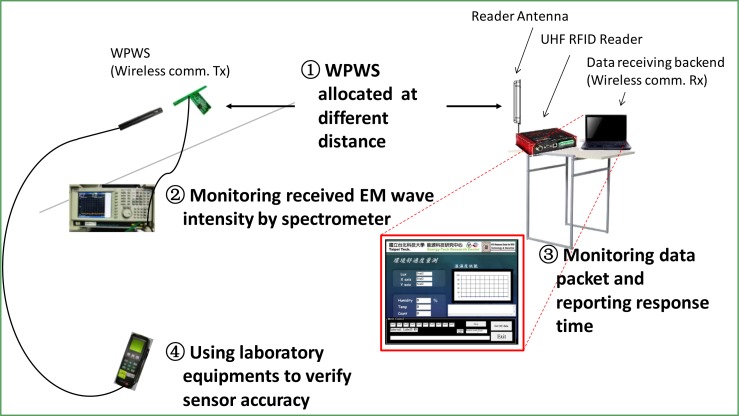
Experimental Setup 1: Four steps were followed to test two performance indices of the WPWS: stability of harvesting energy, and sensor accuracy.

Using hardware, software, and laboratory equipment, this study tests two performance indices of the WPWS: Stability of harvesting energy from EM waves; Accuracy of sensor on the WPWS. Figure 14 shows the experimental setup.

As Figure 14 shows, the WPWS was placed at a distance of 0.5 to 5 m from the UHF RFID reader. The WPWS was powered by EM waves emitted from the reader antenna. The received power intensity was analyzed by a spectrometer. The dB value is reported to determine the stability of harvesting energy. Powered by EM waves, the WPWS can send back sensing data through wireless communication. Wireless comm., Tx, is on WPWS and Rx is inserted into a computer as a data receiving backend. This computer was also used to control the RFID reader and monitor the data packets of the wireless communication. Through packet checking and verifying the error code, the data packet loss rate was controlled at ±1%. Under this packet loss rate, the response time of WPWS equipped with different sensor module is reported. The response time is an important index of the working stability of the sensor node. These data are plotted corresponding to distance. This study uses the dB value of the EM wave power intensity to evaluate the stability of WPWS using harvested energy. Regarding sensor accuracy, the laboratory equipment mentioned in previous sections was used for comparative tests. The deviation between the WPWS data and those from laboratory equipment was plotted by measurement error (%) defined as:

(2) $$\text{Measurement error}\;(\%)=\frac{\left|\text{WPWS data}-\text{Laboratory equipment data}\right|}{\text{Laboratory equipment data}}\times 10$$

**Figure 15.**
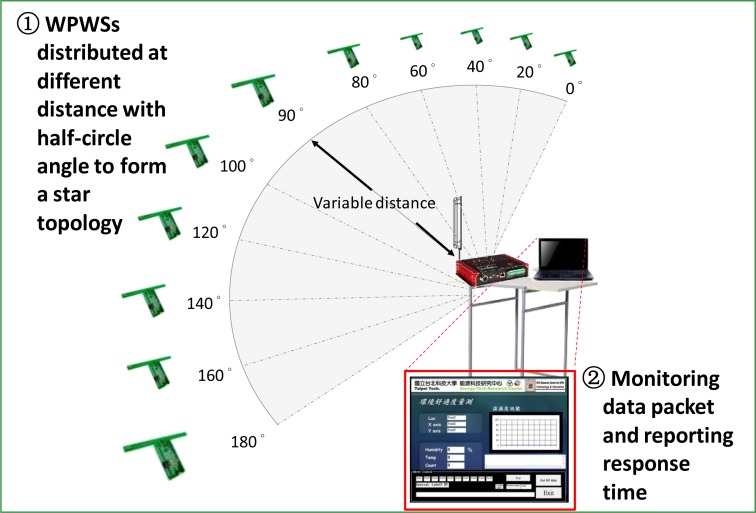
Experimental Setup 2: WPWSs distributed as a star topology, the simplest topology, for tests with two steps.

**Figure 16.**
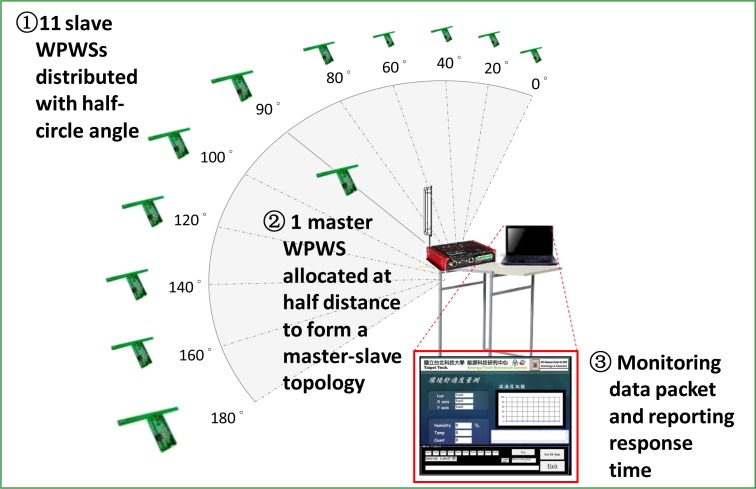
Experimental Setup 3: One WPWS works as a master node and another 11 WPWSs are slave nodes. This arrangement forms a master-slave topology, which is fundamental to constructing a complex sensor network. Three steps are suggested to test.

Another critical performance index of WPWS is the topology for constructing a sensor network. This index is tested by the experimental setups shown in Figures 15 and 16. As shown in Figures 15 and 16, two topologies using WPWS were tested. Figure 15 shows a star topology, which is the simplest form for constructing a sensor network. First, this study tests whether the WPWS can be used to form a star topology. Two steps can be used to test if the WPWS can form a star topology to construct a sensor network.

Unlike the distance test shown in Figure 14, the star topology indicates the distribution range of the WPWSs. A complex topology is needed to construct a sensor network. A master-slave topology is fundamental to constructing a variable topology. Whether the WPWS can work well to form a sensor network with a complex topology is the second issue for testing. Three steps shown in Figure 16 are suggested for test.

The experimental apparatus and setups illustrated above are used to test if WPWS can satisfy the three performance indices described in the Introduction section, also mentioned in this section. Stability is indicated by response time. WPWS should send back sensing data within a reasonable time period. Sensor accuracy is compared with laboratory equipments. Discrepancy (*i.e.*, two data error in %) is reported. These two indices only illustrate if the wireless sensors can be enabled by wireless power. To be a well-functioning sensor node, WPWS should work under a certain topology to form a sensor network. This study tests two topologies: star and master-slave. A star is the simplest type to form a network. Master-slave is the fundamental example of a complex type network. Related results will be reported and discussed in the following section.

## 5. Results and Discussion

**Figure 17.**
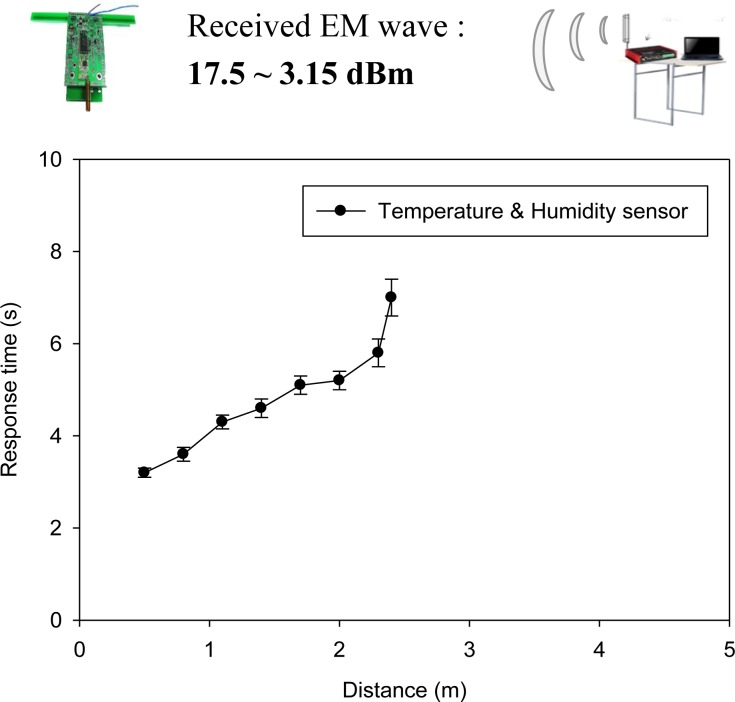
The response time of a WPWS equipped with temperature and humidity sensors.

With respect to the WPWS equipped with different chip-type sensors, the results of experimental Setup 1 show the stability and accuracy of the devices under test. Referring to the specifications introduced in Sections 3.3–3.5, the nominal power of our WPWS equipped with temperature and humidity sensor is approximately 83.18 mW. Under 1 W emitted power from a UHF RFID reader, this device can be enabled from 0.5 to 2.4 m away. Received EM wave power reported by the spectrometer ranges from 17.5 to 3.15 dBm, dB unit with mW base. Data can be sent back continuously. Monitoring software reports the longest response time is around 7 s. The response time can be shortened as the WPWS position approaches the reader. The fastest response time is 3.2 s. Figure 17 plots the response time with respect to different charging distances.

**Figure 18.**
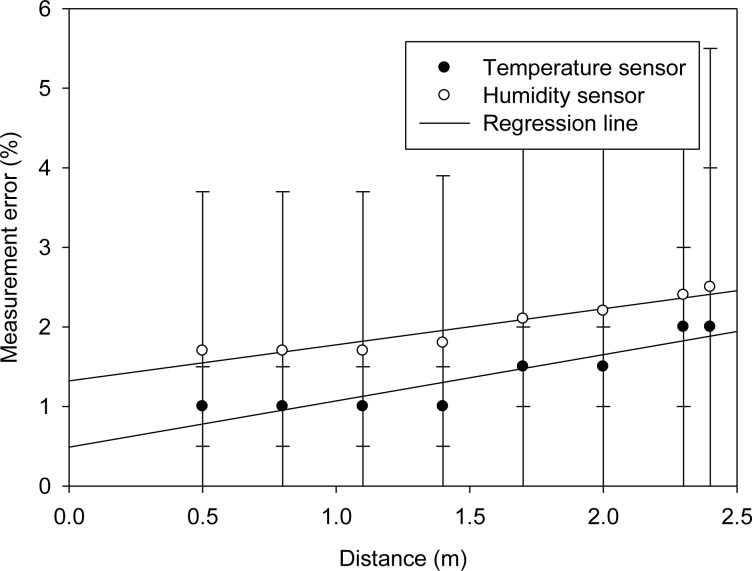
Measurement error of a WPWS equipped with temperature and humidity sensors.

Figure 18 shows the deviations between data obtained from the WPWS equipped with temperature and humidity sensors and those obtained by laboratory equipment. Although a noticeable uncertainty is indicated by error bars, the WPWS can achieve less than 2% error in measuring temperature and humidity.

Table 3 in Section 3.5 shows that, under nominal power supply conditions, a chip-type sensor for measuring temperature and humidity, SHT-10, achieves a 2% and 3% accuracy, respectively. The measurement error shown in Figure 18 indicates that the WPWS provides similar accuracy to battery-powered sensors.

**Figure 19.**
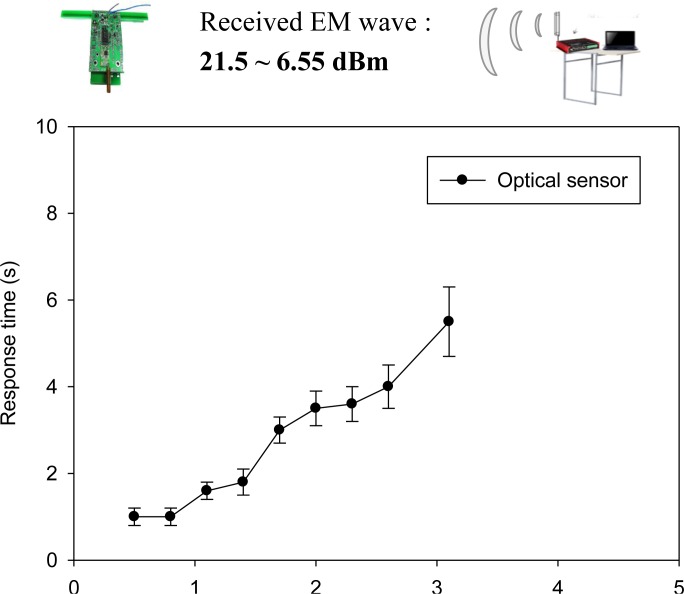
Response time of a WPWS equipped with optical sensor.

Referring to the specifications described in Section 3.6, the nominal power of a WPWS equipped with an optical sensor is approximately 34.68 mW. This is only 40% of the power consumption of a WPWS equipped with temperature and humidity sensors. Under the same driving conditions, the charging distance can be increased to 3.1 m. The received EM wave power reported by the spectrometer ranges from 21.5 to 6.55 dBm. Data can be sent back continuously. Monitoring software reports the longest response time is around 4 s. The fastest response time is 1 s. Figure 19 plots the response time with respect to charging distance.

**Figure 20.**
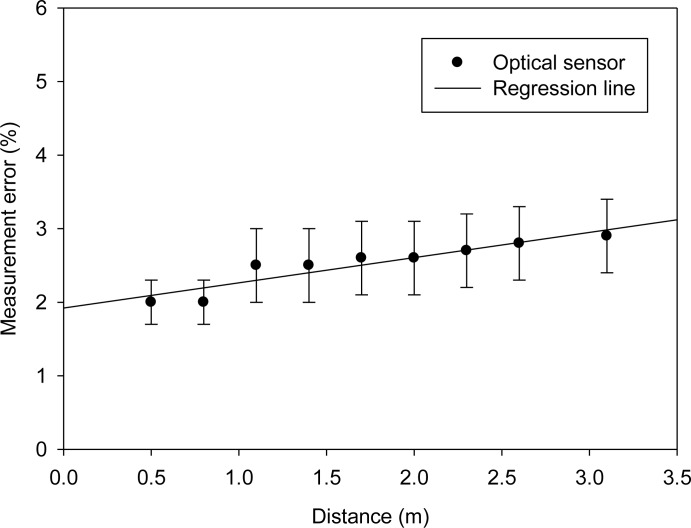
Measurement error of a WPWS equipped optical sensor.

Figure 20 plots the error of a WPWS equipped with an optical sensor. Comparing with the temperature and humidity sensor, the optical one has similar accuracy within 2%–3% deviation from laboratory equipment. It has approximately 4–6 lux accuracy. However, Table 4 in Section 3.6 shows that the optical sensor provides accuracy to ±0.5 lux. WPWS has worse accuracy then those powered by batteries. Unlike the temperature and humidity sensor, the optical sensor is an analog sensor and its signal is processed by the signal processor on the WPWS. Wireless charging may provide limited power to the processor, leading to poor accuracy.

**Figure 21.**
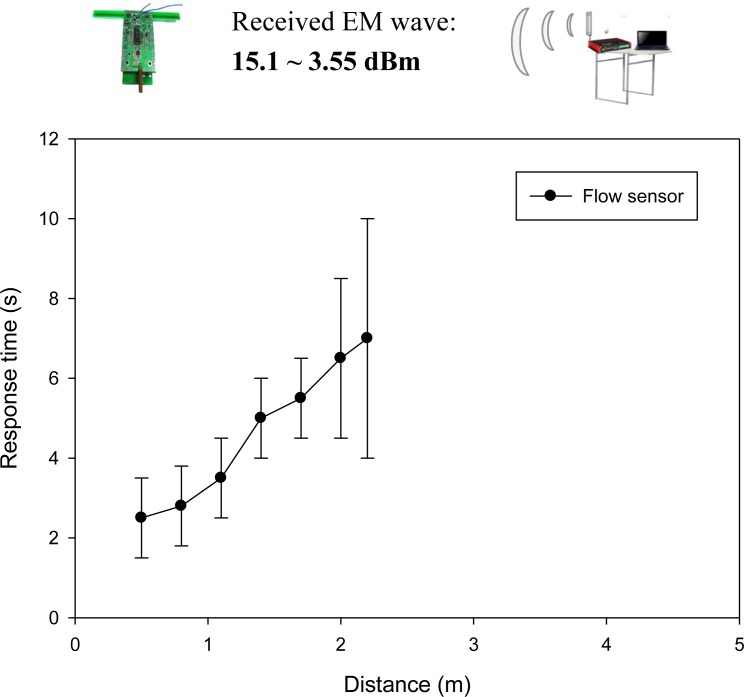
Response time of a WPWS equipped with flow sensor.

The WPWS equipped with a home-made flow sensor has the largest power consumption of 103 mW among the three sensor modules developed in this study. Under the same driving conditions, the farthest charging distance is only extended to 2.2 m. The received EM wave power reported by the spectrometer ranges from 15.1 to 3.55 dBm. Data can be sent back continuously. Monitoring software reports the longest response time is 7 s. The fastest response time is 2.5 s. Although the high-power, WPWS equipped with flow sensor has similar performances compared with the one equipped with temperature and humidity sensor. Figure 21 plots the response time with respect to charging distance.

**Figure 22.**
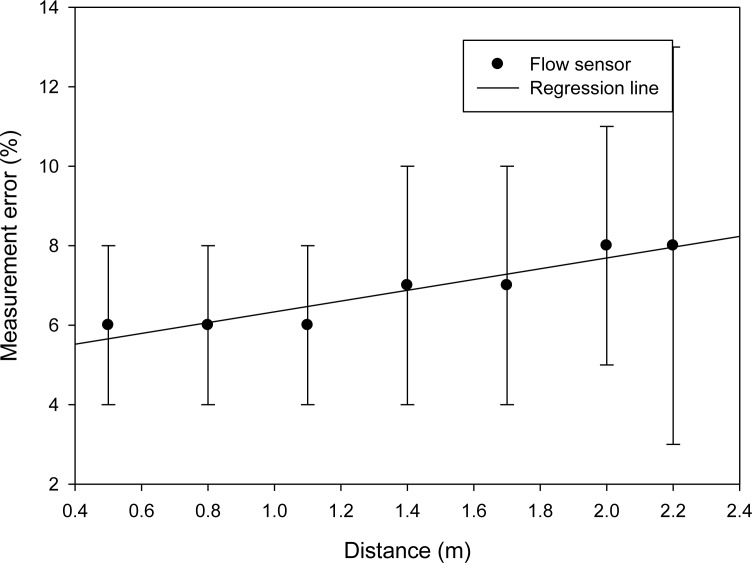
Measurement error of a WPWS equipped with a flow sensor.

Figure 22 shows the measurement error with respect to wireless charging distance. The home-made flow sensor powered by battery provides a ±0.02 m/s accuracy, as shown in previous research [15]. The flow sensor on WPWS had a 0.024 m/s accuracy. The comparative data illustrates that the flow sensor can work well with a wireless power supply. However, the WPWS equipped with home-made flow sensor has worse accuracy than the other two types. This may be because high power consumption means that wireless charging provides only limited power to the sensor and signal processer. These limitations yield low accuracy in airflow sensing.

Experimental Setup 1, as shown in Figure 14, shows the results of working stability and sensor accuracy of WPWS reported from Figures 17 to 22. Three WPWS equipped with an optical sensor, temperature and humidity sensor and airflow sensor have power consumptions of 34.68 mW, 83.18 mW and 103 mW. Using a 1 W emitted EM wave, WPWSs received power intensity varies from 21.5 dB, 17.5 dB to 15.1 dBm at 915 MHz. This is because different loadings on the power receiver cause variable received power intensity. These power intensity data reported by the spectrometer show more details about wireless charging. The charging status of the WPWS was evaluated using time-slot FFT. However, we did not perform experiments in an anechoic room, and reflecting EM waves may have caused interference in the spectrometer readings. This in turn creates an obstacle to data analysis in the frequency domain. The response time and time domain analysis result were used to evaluate if WPWS can harvest energy stably from EM waves to drive sensors. For WPWSs equipped with three different sensors and having power consumptions of 34.68 mW, 83.18 mW, and 103 mW, monitoring software captured communication packets at 433 MHz band, checked the accuracy of packets, and reported a response time ranging from 1 s to 7 s. These response times indicate WPWSs work stably and can be an effective tool for detecting the environment.

A comparison of the measurement error of the WPWS and laboratory equipment indicates good accuracy, especially for sensing accuracy achieved by wireless charging. For the WPWSs with power consumptions of 34.68 mW, 83.18 mW, and 103 mW, measurement error percentages range from 2% to 7%. In industrial applications, an 8% error rate is considered acceptable.

The greatest charging distances were 3.1 m, 2.4 m, and 2.2 m for WPWSs with power consumptions of 34.68 mW, 83.18 mW, and 103 mW, respectively. As expected, a higher power consumption yields shorter charging distances. However, this study only tested prototypes with three different power consumption levels, making it difficult to derive a relationship between wireless charging distance and power consumption. More research is necessary to investigate the details of wireless charging.

Instead of a detailed investigation of wireless charging, this study focuses on developing a wireless sensor enabled by wireless power. Using a standard RFID reader, the WPWS can be enabled as far as to 2 m away. As Figure 14 shows, test results were obtained only because the WPWS was placed in front of the RFID antenna. However, to form a sensor network, WPWSs should be distributed not just in front of the reader. Subsequently, experimental Setup 2 and 3 shown in Figures 15 and 16, respectively, were arranged to test the ability of WPWS to construct a sensor network.

**Figure 23.**
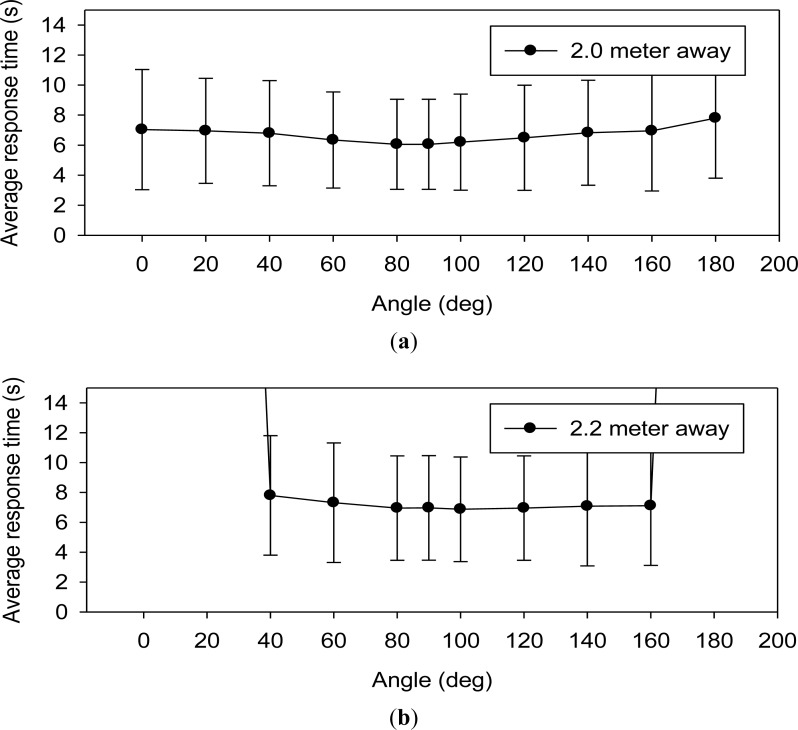

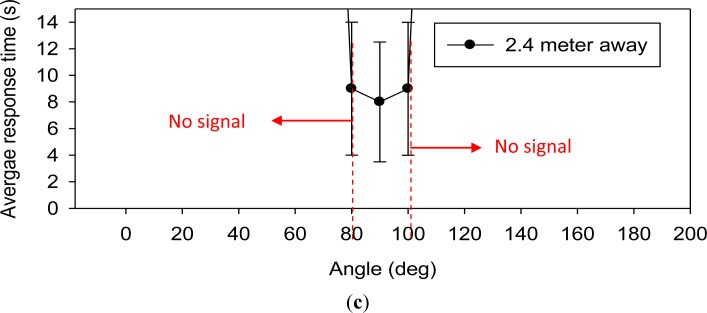
TheWPWSs can form a star topology to construct a sensor network. The average response time of 11 WPWSs distributed at 2 m, 2.2 m and 2.4 m diameter area appear in (**a**), (**b**), and (**c**) respectively.

The experimental Setup 2 shown in Figure 15 indicates that the WPWSs can form a star topology, which is the simplest topology for constructing a sensor network. In this star topology, 11 WPWSs are distributed in a half-circle angle at a remote distance. These WPWSs send back data to a central computer equipped with wireless comm., Rx. A standard UHF RFID reader is also positioned centrally. Within a 2 m to 2.4 m diameter, 11 WPWSs equipped with three different sensor modules were powered by EM wave emitted from the RFID reader and were able to send back data. Figure 23 shows the response time and the average data for different sensor modules.

Figure 23 shows the test results of a star topology constructed by WPWSs. Referring to panels (a), (b) and (c), all WPWSs can send back signals only within a 2.0 m diameter area. Beyond this range, some devices lose the signal, especially those positioned at a wide angel range. At 2.4 m, only three WPWSs positioned just in front of the RFID reader (within 20° angle) can send back signals. Although wireless charging can power a WPWS from up to 3.1 m away, this star network topology only allows a 2.0 m working range for the sensor network.

**Figure 24.**
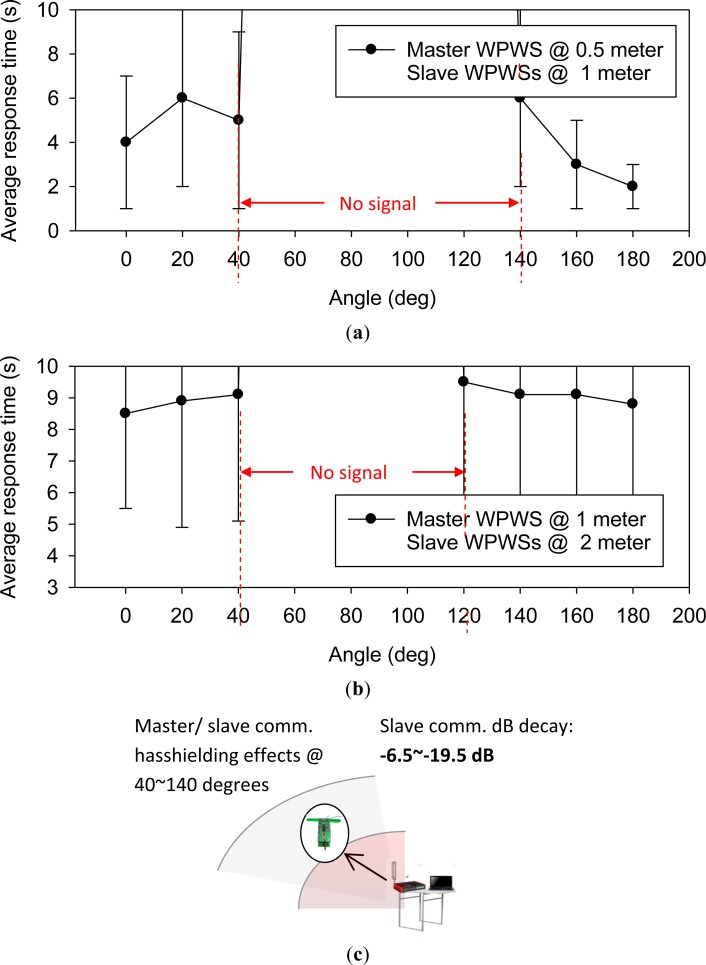
The WPWSs can form a star topology to construct a sensor network. The average response time of one master WPWS located at 0.5 m and 1 m and 11 WPWSs distributed at 1 m and 2 m diameter area appear in (**a**) and (**b**). The dB decay with and without master WPWS appears in (**c**).

This study also investigated a master-slave topology. The experimental Setup 3 shown in Figure 16 shows the test method. In a master-slave topology, each WPWS located in the outer area sends back data to one WPWS near the center. This WPWS is called the master WPWS, and delivers all data to the central data receiving backend. In this study, we modified one WPWS and equipped it with a wireless comm., Rx, to obtain data from another 11 WPWSs. Table 2 in Section 3.4 shows that the Rx module has power consumption similar to a WPWS with only one Tx. Thus, the master WPWS can be easily enabled by a RFID reader. Because of limited memory on master WPWS, we kept the RS 232 port on the Rx and sent back signals from 11 slave WPWSs to a central computer by line communication. The master WPWS was positioned at 0.5 m or 1 m away, and the slave WPWSs were positioned in a half angle range at 1 m or 2 m. Three different sensor modules produced different response times, and Figure 24 plots the average data. Except reporting average response time, Figure 24 reports the dB decay of the slave WPWSs. This figure indicates changes in the received EM wave intensity with or without master WPWS.

Figure 24(a) shows that slave WPWSs distributed from 40 to 140 degrees lose signal because they are behind the master WPWS. Test results indicate that a strong shielding effect occurs when WPWSs are arranged to form a master-slave topology. Figure 24(b) shows that more WPWSs can send back signals when the whole topology is moved away from 1 m to 2 m. This indicates that the shielding effect is more significant when the topology is closer to the EM wave source. As Figure 24(c) shows, the decay in the received EM wave power reaches −6.5 dB to −19.5 dB. This significant signal decay yields WPWSs cannot be used to form a master-slave topology for constructing a sensor network.

In summary, the three experimental setups in this study test three performance indices of the WPWS. The first index is the energy harvesting stability. Experimental results show that the WPWS can stably harvest power from a standard UHF RFID reader and send back signals within 8 s. The wireless charging distance varies from 2.2 m to 3.1 m for WPWSs with power consumption levels ranging from 103 to 34.68 mW. The second index is the accuracy of the sensors on the WPWS. Results show that WPWSs equipped with three different sensors, including temperature and humidity, optical, and air velocity, can provide sensing data as accurate as those obtained from laboratory equipment. Measurement errors of all test conditions are within 8%. The last index is to construct a sensor network using WPWSs. Results show that the WPWS can form a star topology, the simplest topology. However, the WPWSs cannot form a master-slave topology because of the shielding effect of wireless charging.

## 6. Conclusions

This study investigated the feasibility of a wireless sensor powered wirelessly. Specifically, this study proposes a dual antenna structure to harvest energy from EM waves at 915 MHz and send data through wireless communication at 433 MHz. A wireless power driven wireless sensor, called a WPWS, works just like a RFID passive tag with no need for battery power. Unlike an RFID tag, which can only be queried, the WPWS has a complete three-layer topology to fulfill the function of a wireless sensor node. Dual working bands forma special PHY layer. This is the first report of a sensor node that can harvest power and communicate wirelessly. Following the Modbus protocol, each data packet has a MAC layer, facilitating the delivery of sensor data to the backend. The Modbus protocol also supports the object definition of the APP layer. This topology definition at the APP layer enables the WPWS to construct a sensor network.

The special PHY layer creates uncertainties when working with the WPWS. This study tests the stability of harvesting energy as the first performance index. Experimental results show that different WPWS designs have power consumptions ranging from 30 to 100 mW at distances of 3.1 m to 2.4 m. Data can be sent back continuously, and the response time is within 8 s. Thus, WPWS can work stably for sensing environment and send back data in a reasonable time.

The accuracy of data is the second issue. This study compares the data detected from different WPWSs equipped with temperature and humidity, optical, and airflow velocity sensors with those obtained by laboratory equipment. The deviation is within 8%. This type of data discrepancy is acceptable for industrial applications.

The topology formed by the WPWS for constructing a sensor network is the last issue, and remains an important topic for future research. Experimental results show that the WPWS can only form a star topology, the simplest topology for sensing an environment. Using a standard UHF RFID reader, a star topology can extend the measurement area to 2 m and collect data from 11 WPWSs distributed in a half-circle angle. Using two opposite antennas attached to one reader, a 4 m area can be monitored using WPWSs. For a complex topology with master-slave communication, the shield effect creates a significant decay in power receiving on slave WPWSs placed behind the master WPWS. This prevents using WPWSs to construct a sensor network with a complex topology. Thus, more research is necessary to develop a wireless sensor network enabled by wireless power.

Despite its limitations, this development of the WPWS still helps extend sensor network applications. A star topology can be used to extend the measuring area from a root node with power. By harvesting power from EM waves emitted from root node, the distributed WPWS needs no batteries. This greatly increases the reliability of sensor nodes, and is most suitable for applications in hazard environments or special military applications.

## Figures and Tables

**Table 1.** Parameters of the signal processor.

**Parameter**	**Value or Index**
Computing speed	5 MIPS
Data communication	Serial protocol interface (SPI) or I2C

Analog to digital conversion (ADC)	12 channels with 10 bits resolution
Clock frequency	20 MHz
Working voltage	2.0 to 5.5 V
Power consumption	1.8–4.5 mA @ 2.0–5.5 V Nominal power − 14.85 mW

**Table 2.** Parameters of the wireless communication module.

**Parameter**	**Value or index**
Communication speed	1.2–500 kbps
Communication distance	Up to 350 m
Data coding method	FSK/OOK/ASK/MSK FSK suggested by ISO 18000-7 is used in this study
Packet loss rate	±1% Using LQI
Working voltage	1.8 to 3.6 V
Power consumption	Tx < 25 mA Rx < 15 mA Nominal power for Tx − 18 mW For Rx − 11 mW

**Table 3.** Parameters of the temperature the humidity sensor module.

**Parameter**	**Temperature**	**Relative humidity**
Detection range	−40 °C–+123 °C	0%–100%
Accuracy	±0.5 °C, at 25 °C	±3% RH (20%–80% RH)
Response time	≤2 s	≤4 s
Resolution	±0.01 °C	0.03% RH
Working voltage	2.4 V–5.5 V	
Power consumption	10–30 mA@2.4–5.5 V Nominal power − 50 mW	

**Table 4.** Parameter list of the optical sensor module.

**Parameter**	**Value or Index**
Sensitivity	2 Ω/lux
Accuracy	±0.5 lux
Response time	30 msec
Resolution (using ADC embedded in signal processor)	0.01 lux
Working voltage	2.5–5 V
Power consumption	1.5 mW

**Table 5.** Parameters of the flow sensor module.

**Parameter**	**Value or index**
Sensitivity	0.5 mV m^−1^·sec^−1^
Accuracy	±0.02 m/sec
Response time	30 ms
Resolution (using ADC embedded in signal processor)	0.001 m/s
Working voltage	2.5–5 V
Power consumption	70 mW
